# Physiological and transcriptomic analyses reveal the roles of secondary metabolism in the adaptive responses of *Stylosanthes* to manganese toxicity

**DOI:** 10.1186/s12864-020-07279-2

**Published:** 2020-12-03

**Authors:** Yidan Jia, Xinyong Li, Qin Liu, Xuan Hu, Jifu Li, Rongshu Dong, Pandao Liu, Guodao Liu, Lijuan Luo, Zhijian Chen

**Affiliations:** 1 Institute of Tropical Crop Genetic Resources, Chinese Academy of Tropical Agricultural Sciences, Haikou, 571101 China; 2 Hainan Key Laboratory for Sustainable Utilization of Tropical Bioresources, College of Tropical Crops, Hainan University, Haikou, 570110 China; 3 College of Biology and Pharmacy, Yulin Normal University, Yulin, 537000 China

**Keywords:** *Stylosanthes*, Manganese toxicity, Oxidative stress, Secondary metabolism, Transcription factor, Transcriptomics, Heavy metal

## Abstract

**Background:**

As a heavy metal, manganese (Mn) can be toxic to plants. Stylo (*Stylosanthes*) is an important tropical legume that exhibits tolerance to high levels of Mn. However, little is known about the adaptive responses of stylo to Mn toxicity. Thus, this study integrated both physiological and transcriptomic analyses of stylo subjected to Mn toxicity.

**Results:**

Results showed that excess Mn treatments increased malondialdehyde (MDA) levels in leaves of stylo, resulting in the reduction of leaf chlorophyll concentrations and plant dry weight. In contrast, the activities of enzymes, such as peroxidase (POD), phenylalanine ammonia-lyase (PAL) and polyphenol oxidase (PPO), were significantly increased in stylo leaves upon treatment with increasing Mn levels, particularly Mn levels greater than 400 μM. Transcriptome analysis revealed 2471 up-regulated and 1623 down-regulated genes in stylo leaves subjected to Mn toxicity. Among them, a set of excess Mn up-regulated genes, such as genes encoding PAL, cinnamyl-alcohol dehydrogenases (CADs), chalcone isomerase (CHI), chalcone synthase (CHS) and flavonol synthase (FLS), were enriched in secondary metabolic processes based on gene ontology (GO) analysis. Numerous genes associated with transcription factors (TFs), such as genes belonging to the C2H2 zinc finger transcription factor, WRKY and MYB families, were also regulated by Mn in stylo leaves. Furthermore, the C2H2 and MYB transcription factors were predicted to be involved in the transcriptional regulation of genes that participate in secondary metabolism in stylo during Mn exposure. Interestingly, the activation of secondary metabolism-related genes probably resulted in increased levels of secondary metabolites, including total phenols, flavonoids, tannins and anthocyanidins.

**Conclusions:**

Taken together, this study reveals the roles of secondary metabolism in the adaptive responses of stylo to Mn toxicity, which is probably regulated by specific transcription factors.

**Supplementary Information:**

The online version contains supplementary material available at 10.1186/s12864-020-07279-2.

## Background

Of the mineral nutrients, manganese (Mn) is essential for plant growth and participates in a series of metabolic processes, such as photosynthesis, respiration, secondary metabolism and protein biosynthesis [1]. Mn acts as a cofactor of many enzymes, such as superoxide dismutase (SOD) and enzymes involved in the tricarboxylic acid cycle. Mn also plays roles in flavonoid and lignin biosynthesis [2]. As a trace element, Mn is only required in small amounts of 20–40 mg Mn per kilogram dry weight for most plants [3]. However, Mn is also considered to be a heavy metal that can cause phytotoxicity when it reaches the level of 150 mg per kilogram dry weight in plants [1].

In soils, available Mn levels fluctuate from 450 to 4000 mg per kilogram, and Mn solubility is mainly dependent on pH values and redox conditions [4, 5]. Hence, excess Mn toxicity generally occurs in acid soils due to the accumulation of bioactive divalent Mn (II) [5, 6]. Consequently, soil amelioration, such as lime application, is typically conducted to alleviate Mn toxicity by decreasing Mn availability, but this application is costly from both economic and environmental aspects [7]. For these reasons, breeding crop varieties with superior Mn tolerance represents a sustainable alternative agronomical strategy, but this strategy requires better understanding of how plants respond to Mn toxicity. Although morphological changes in plants grown under the condition of Mn toxicity vary among plant species, the appearances of Mn toxicity reported in most plants generally include leaf chlorosis, brown spots, crinkled leaves and brown roots, and ultimately plant growth inhibition [8, 9]. Adverse impacts caused by Mn toxicity have also been documented in plant cells at physiological levels, such as triggering oxidative stress, causing lipid peroxidation, inhibiting enzyme activity, impairing chlorophyll biosynthesis and photosynthesis and disturbing the uptake and translocation of other mineral elements [1, 9].

To counteract Mn toxicity, plants are equipped with sophisticated adaptive strategies to detoxify Mn, such as modified Mn translocation and distribution, sequestration of Mn into subcellular compartment, antioxidant system activation, and adjusted root organic acid exudation [9]. Cumulative results show that reactive oxygen species (ROS) scavenging systems involving antioxidant enzymes, including peroxidase (POD) and ascorbate peroxidase (APX), are regulated by the plant’s response to Mn toxicity, thereby alleviating excess Mn-induced oxidative stress [10, 11]. Furthermore, secondary metabolic processes and metabolites, such as phenolics, flavonoids and phenylalanine, are regulated by Mn stress in plants [2, 12, 13], suggesting the potential roles of secondary metabolism in the adaptation of plants to Mn toxicity. To date, some key genes have been characterized that participate in Mn uptake, translocation and distribution and help plants address environmental Mn stress [14, 15]. For example, as one of the natural resistance-associated macrophage protein (Nramp) members, OsNramp3 in rice (*Oryza sativa*) is a plasma membrane Mn transporter and responsible for Mn distribution from young leaves and panicles to old tissues, thereby protecting plants from Mn toxicity [16]. Metal tolerance proteins (MTPs) belonging to the cation diffusion facilitator (CDF) family, such as ShMTP1 from the Caribbean stylo (*Stylosanthes hamata*) [17], OsMTP8.1 and OsMTP8.2 from rice [18, 19], AtMTP8 from *Arabidopsis* [20], CsMTP8 from cucumber (*Cucumis sativus*) [21] and CasMTP8 from the tea plant (*Camellia sinensis*) [22], are involved in sequestering Mn into the vacuole for detoxification. Although the roles of the above genes have been functionally characterized, the transcriptome profiles of Mn-responsive genes in plants have not been fully elucidated. Studying the responses of plants to varying Mn concentrations is useful to determine how plants cope with Mn toxicity.

Stylo (*Stylosanthes* spp.) is a dominant tropical legume that is widely grown in tropical areas worldwide [23, 24]. Superior Mn tolerance is observed in the stylo compared to other legumes [25]. Recently, it has been documented that high Mn adaptability in stylo may be achieved by its fine regulation of proteins involved in specific pathways, such as defense response, photosynthesis and metabolism [11]. Furthermore, important roles of organic acids, such as malate, in stylo adaptation to Mn toxicity have been reported; a malate dehydrogenase (SgMDH1) enzyme that is up-regulated in response to Mn catalyzes malate synthesis and contributes to Mn detoxification [26]. Although stylo has considerable potential for Mn tolerance, the effects of excess Mn toxicity on profile alterations in the gene expression of stylo have not been reported, and the molecular responses of stylo to Mn stress remain largely unknown. Previous studies have paved the way for the current study dissecting the molecular responses of stylo to Mn toxicity. Accordingly, in this study, the effects of various Mn concentrations on the physiological changes in stylo were first investigated. Transcriptomic analysis of Mn-responsive genes in stylo leaves was further performed using an RNA-seq approach. The results of this study provide a platform to understand the adaptive responses of stylo to Mn toxicity and the genes involved.

## Results

### Effects of excess Mn stress on stylo growth

**Fig. 1:**
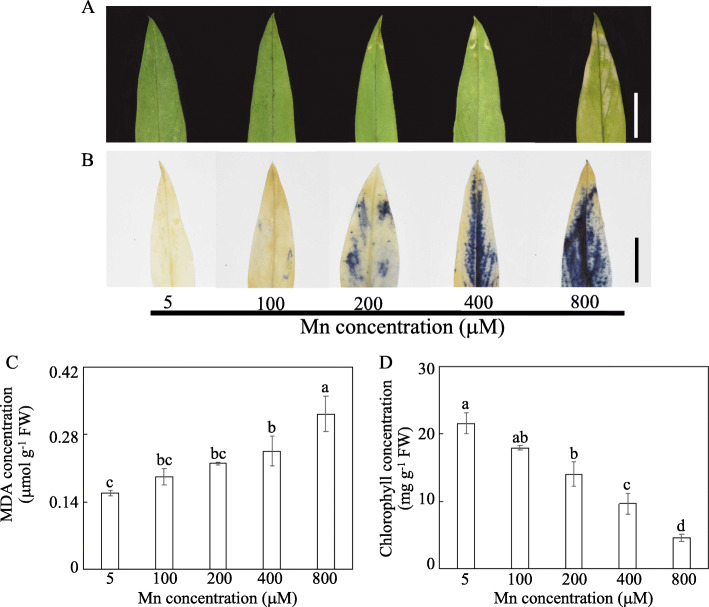
Effects of different Mn treatments on stylo growth. **a** Stylo leaves with different Mn treatments. **b** NBT staining of stylo leaves treated with different Mn concentrations. **c** MDA concentrations in leaves. **d** Chlorophyll concentrations in leaves. Thirty-day-old stylo plants were treated with 5, 100, 200, 400 and 800 μM MnSO_4_ for 10 d. Values are the mean of three replicates with standard error bars. Different letters represent significant differences at *P* < 0.05. Bar = 1 cm

Thirty-day-old stylo plants were subjected to Mn treatments ranging from 5 to 800 μM MnSO_4_ for 10 d. Leaf chlorosis, a symptom of Mn toxicity, was observed in stylo leaves treated with greater than 200 μM Mn, especially 400 and 800 μM Mn (Fig. 1a). O_2_^−^ levels were observed in leaves based on nitroblue tetrazolium chloride (NBT) staining (Fig. 1b). O_2_^−^ accumulation was mainly observed in leaves treated with excess Mn compared with the control (5 μM), and the most intense blue color was observed in leaves treated with 800 μM Mn (Fig. 1b). Consistent with this finding, increases in malondialdehyde (MDA) levels were observed in stylo leaves subjected to high Mn stress. MDA concentrations in leaves exposed to 400 and 800 μM Mn treatments were 55.4 and 103.9% greater, respectively, compared with control conditions (Fig. 1c). Furthermore, the relative electrolyte leakage of stylo leaves was significantly increased under Mn treatments exceeding 200 μM (Additional file 1: Fig. S1). In contrast, leaf chlorophyll concentrations were decreased by 35.0–79.1% upon treatment with 200 to 800 μM Mn compared with the control (Fig. 1d). Similarly, the maximum quantum yield of photosystem II (*F*v/*F*m) significantly declined in stylo under Mn treatments from 200 to 800 μM Mn compared with the controls (Additional file 1: Fig. S1), suggesting that photosynthesis was inhibited by Mn toxicity.

**Fig. 2:**
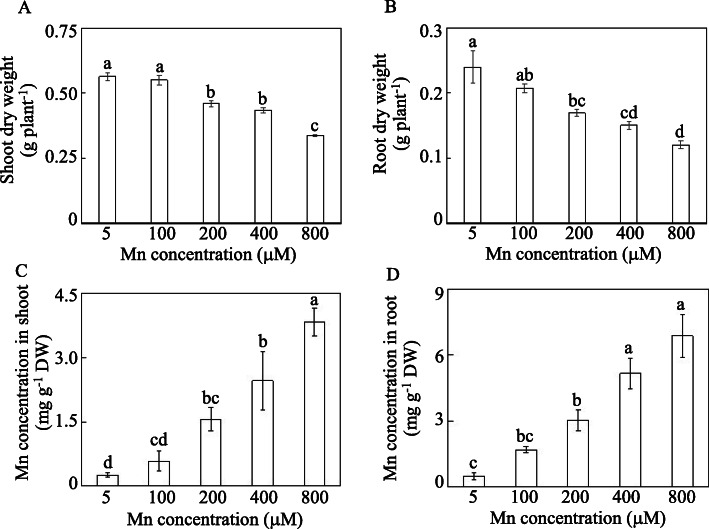
Plant dry weight and Mn concentrations in stylo under different Mn treatments. **a** Shoot dry weight. **b** Root dry weight. **c** Shoot Mn concentrations. **d** Root Mn concentrations. Thirty-day-old stylo plants were treated with 5 to 800 μM MnSO_4_ for 10 d. Values are the mean of three replicates with standard error bars. Different letters represent significant differences at *P* < 0.05

Stylo shoot and root growth were inhibited by Mn concentrations greater than 200 μM. The shoot dry weight was reduced by 29.4–50.0% with 200 to 800 μM Mn treatments, whereas root dry weight decreased by 18.3–40.2% with 200 to 800 μM Mn treatment compared with their respective controls (Fig. 2a, b). Additionally, stylo plant height decreased at Mn levels greater than 400 μM compared with the controls (Additional file 1: Fig. S1). Increases in Mn concentrations were found in both stylo shoots and roots under Mn stress. Mn concentrations in shoots and roots were increased by more than 2.2-fold and 3.5-fold with more than 100 μM Mn compared with their respective controls (Fig. 2c, d).

### Enzyme activity response to excess Mn

**Fig. 3:**
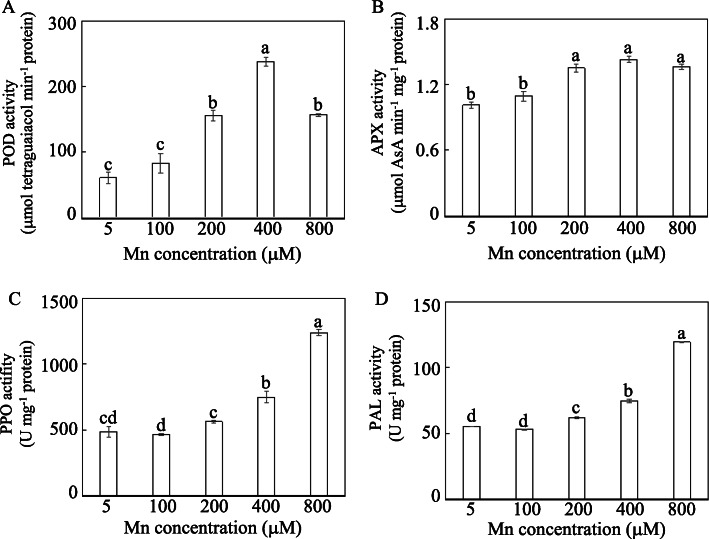
Determination of enzyme activities. **a** POD activity. **b** APX activity. **c** PPO activity. **d** PAL activity. Thirty-day-old stylo plants were treated with 5 to 800 μM MnSO_4_ for 10 d. Values are the mean of three replicates with standard error bars. Different letters represent significant differences at *P* < 0.05

Activities of peroxidase (POD), ascorbate peroxidase (APX), polyphenol oxidase (PPO) and phenylalanine ammonia-lyase (PAL) were analyzed in stylo leaves exposed to Mn treatments. The results showed that the tested enzymes were differentially regulated by Mn (Fig. 3). Compared to their respective controls, POD and APX activities increased as Mn treatments increased from 200 to 800 μM, peaking at 400 μM Mn (Fig. 3a, b). POD and APX activities increased by 2.9-fold and 0.41-fold with 400 μM Mn treatment compared to their respective controls (Fig. 3a, b).

In addition, increases in PPO and PAL activities were also found in stylo leaves under Mn stress, especially 400 and 800 μM Mn treatments (Fig. 3c, d). PPO and PAL activities in stylo leaves treated with 400 μM Mn were 54.7 and 35.8% increased compared with their controls (Fig. 3c, d).

### Transcriptome analysis of stylo leaves responding to Mn toxicity

In this study, comparative transcriptomic analysis in stylo leaves subjected to 5 and 400 μM Mn treatments was performed. Approximately 48.4 and 51.6 million clean reads were obtained from the libraries of the stylo leaves treated under control Mn (5 μM) and toxic Mn (400 μM) conditions (Additional file 2: Table S1), respectively. As the genome sequence for stylo is not available, a de novo assembly approach was employed. De novo assembly of the reads produced 234,557 transcripts corresponding to 102,872 unigenes from all samples (Additional file 2: Table S1). The mean lengths of transcripts and unigenes were 1132 and 869 bp, respectively (Additional file 2: Table S1).

**Fig. 4:**
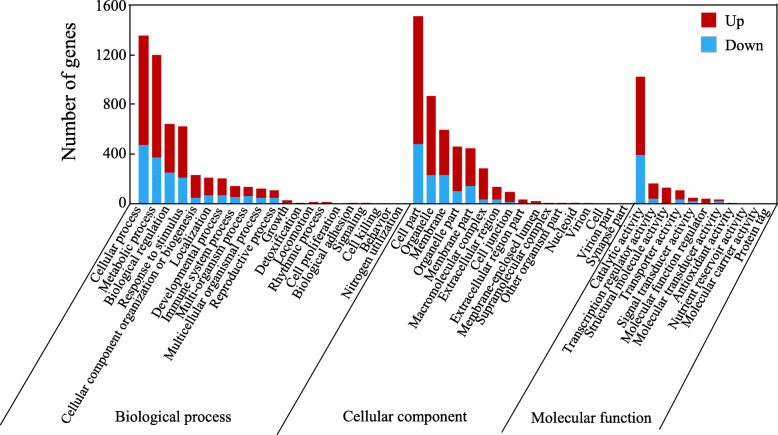
GO analysis of DEGs in stylo. The up or down-regulated genes were classified into biological process, cellular component and molecular function. X- and Y-axis indicate GO terms and the number of DEGs, respectively

**Fig. 5:**
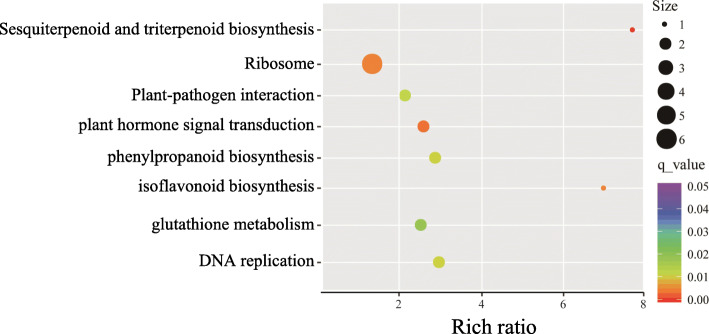
KEGG enrichment analysis of the DEGs. R package ggplot2 was used to data visualization. Rich ratio is the number of significant genes divided by background genes of corresponding pathway term. The size of the dot represents the number of DEGs and the color indicates *q*-value (the corrected *p*-value) of the pathway term

Differentially expressed genes (DEGs) in stylo leaves exposed to Mn treatments were identified based on |log2(fold change)| ≥ 1 and *q* < 0.05. A total of 4094 DEGs were identified via comparison of the two Mn treatments (Additional file 3: Table S2). Among these genes, 2471 genes were up-regulated, while 1623 genes were down-regulated by Mn toxicity (Additional file 3: Table S2). Gene ontology (GO) analysis showed that the identified DEGs can be classified into 21 biological processes (BP), 17 cellular components (CC), and 11 molecular function (MF) terms (Fig. 4). The main categories in BP included cellular process, metabolic process, biological regulation and response to stimulus terms. The dominant categories in CC included cell part, organelle and membrane. Prominent MF categories included catalytic activity, transcription regulator activity and structural molecule activity (Fig. 4). Furthermore, DEGs were mainly involved in the following pathways based on Kyoto Encyclopedia of Genes and Genomes (KEGG) enrichment analysis: ribosome, plant hormone signal transduction, plant-pathogen interaction, glutathione metabolism, phenylpropanoid biosynthesis, DNA replication, sesquiterpenoid and triterpenoid biosynthesis, and isoflavonoid biosynthesis (Fig. 5).

### Secondary metabolic pathways in response to Mn toxicity

**Fig. 6:**
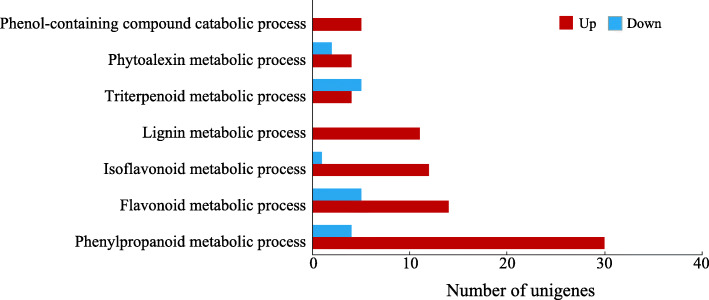
DEGs related to secondary metabolism in stylo’s response to Mn toxicity

**Fig. 7:**
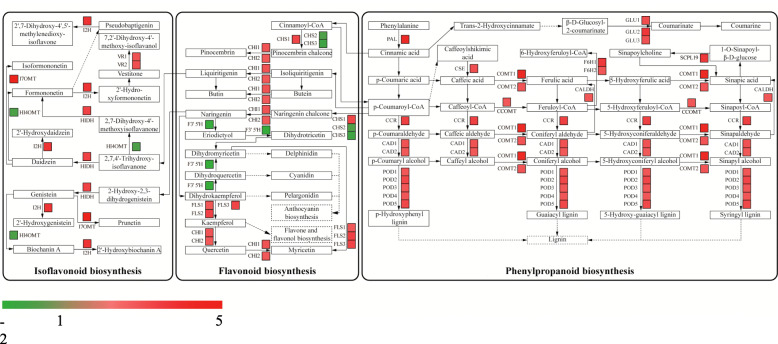
DEGs associated with phenylpropanoid, flavonoid and isoflavonoid biosynthesis pathways. DEGs were mapped to the reference pathways in KEGG. Gene transcripts are presented as a heatmap for log_2_(fold change) of gene transcription between control (5 μM) and Mn toxicity (400 μM) treatments. Gene expression levels range from red (up-regulated) to green (down-regulated). The copyright permission to use and modify these secondary metabolism pathways in the figure has been granted from KEGG

According to GO analysis, a total of 1201 unigenes were predicted to be involved in metabolic processes in the BP categories, including 824 up-regulated unigenes and 377 down-regulated unigenes (Fig. 4). Among these DEGs, a set of 94 genes were enriched in seven secondary metabolic pathways (Additional file 4: Table S3). The top Mn-responsive genes belonged to phenylpropanoid metabolic process, flavonoid metabolic process, isoflavonoid metabolic process and lignin metabolic process (Fig. 6). Interestingly, a large number of genes in the above pathways were enhanced by Mn toxicity. For example, genes encoding PAL, β-glucosidases (GLUs), cinnamoyl-CoA reductase (CCR), cinnamyl-alcohol dehydrogenases (CADs), feruloyl-CoA 6-hydroxylases (F6Hs) and caffeic acid 3-O-methyltransferases (COMTs), were up-regulated in the phenylpropanoid biosynthesis pathway (Fig. 7). In addition, the transcript of genes encoding chalcone isomerases (CHIs), one homolog of chalcone synthase (CHS) and flavonol synthases (FLSs) associated with the flavonoid biosynthesis pathway, isoflavone 7-O-methyltransferase (I7OMT), 2-hydroxyisoflavanone dehydratase (HIDH), isoflavone 2-hydroxylase (I2H) and vestitone reductases (VRs) related to the isoflavonoid biosynthesis process was enhanced in stylo under Mn toxicity (Fig. 7). Furthermore, transcripts of *PAL* and a set of homologs of *I7OMT* and gene encoding anthranilate N-methyltransferase-like in the above pathways were increased by more than 8-fold under Mn stress (Additional file 4: Table S3).

### Transcription factors involved in stylo responses to Mn toxicity

**Fig. 8:**
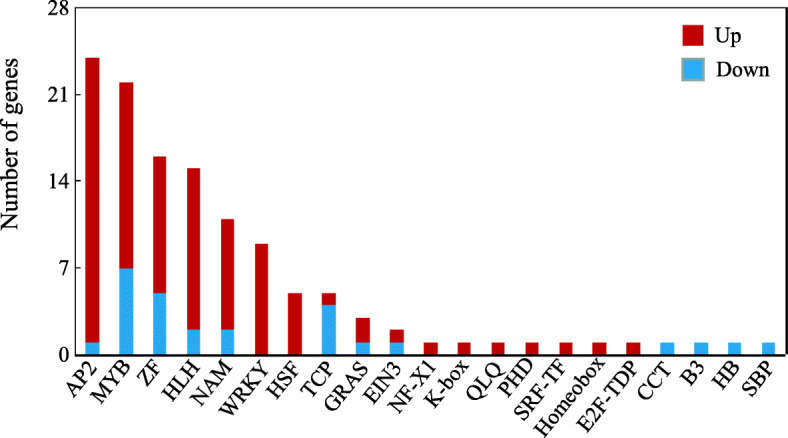
DEGs associated with transcription factors in stylo leaves. X- and Y-axis indicate the abbreviation and the number of TFs, respectively

A total of 123 DEGs were enriched in transcription factors (TFs) (Additional file 5: Tables S4). Among them, the largest group of TFs belonged to the AP2 family with 23 up-regulated and 1 down-regulated genes. Other DEGs encoding TFs included 22 *MYBs*, 16 *ZFs*, 15 *HLHs*, 11 *NAMs*, 9 *WRKYs*, 5 *HSFs*, 5 *TCPs*, 3 *GRASs*, 2 *EINs*, and one of each of the following genes: *NF*-*X1*, *K*-*box*, *QLQ*, *PHD*, *SRF*-*TF*, *Homeobox*, *E2F*-*TDP*, *CCT*, *B3*, *HB* and *SBP* (Fig. 8). Among them, 11 genes were significantly increased by greater than 8-fold under Mn toxicity: *DREB group protein*, *protein PPLZ02*, *dehydration-responsive element-binding protein 1E-like*, *ethylene*-*responsive transcription factor 4* and *TINY transcription factor* belonging to the AP2 family; *myb*-*related protein Zm1* belonging to the MYB family; *zinc finger of C2H2 type* belonging to the ZF family; *transcription factor bHLH18*-*like* belonging to the HLH family; *hypothetical protein LR48* belonging to the WRKY family; *heat shock factor protein HSF30*-*like* belonging to the HSF family; *hypothetical protein GLYMA* belonging to the NF-X1 family, and *MADS*-*box transcription factor 1-like isoform X1* belonging to the K-box family (Additional file 5: Table S4).

### Protein-protein interaction network analysis

**Fig. 9:**
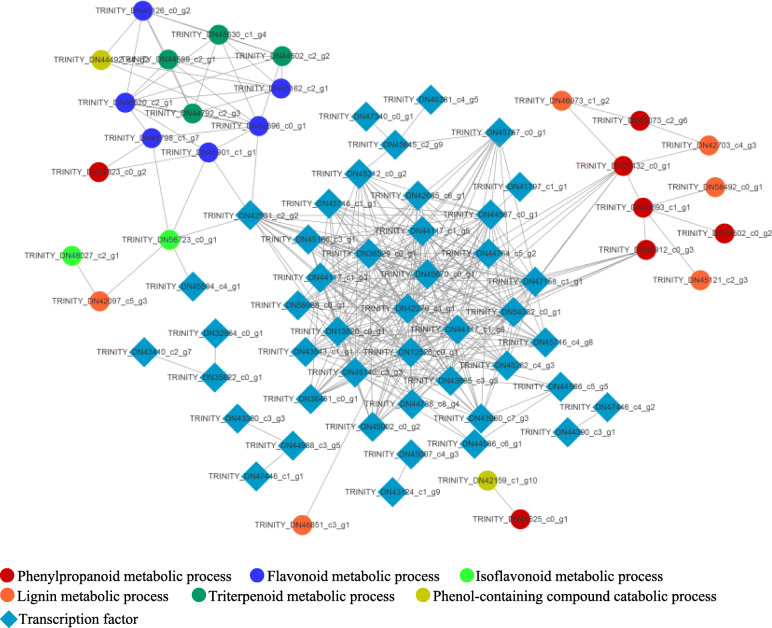
Interaction networks of DEGs related to secondary metabolism pathways and transcription factors. Gene IDs are available in Additional file 4: Table S3 and Additional file 5: Table S4. The interaction networks were constructed using Cytoscape (version 3.8.0). Nodes and edges indicate genes and interactions, respectively

To explore the candidate TFs involved in the transcriptional regulation of genes associated with secondary metabolism in stylo during Mn exposure, the protein-protein interaction networks of DEGs related to secondary metabolic pathways and TFs were further constructed. The interaction networks contained 305 edges with 72 nodes (Fig. 9). Among them, two genes *CAD1*/*2* (TRINITY_DN21432_c0_g1 and TRINITY_DN45912_c0_g3) were regulated by six TFs, all of which belonged to C2H2 zinc finger transcription factors (TRINITY_DN12526_c0_g1, TRINITY_DN42276_c1_g1, RINITY_DN54332_c0_g1, TRINITY_DN13820_c0_g1, TRINITY_DN46140_c3_g3 and TRINITY_DN47158_c1_g1) (Fig. 9). Furthermore, *HIDH* (TRINITY_DN56723_c0_g1) can be regulated by the *MYB* gene (TRINITY_DN42931_c2_g2) and a *NAC-like transcription factor* (TRINITY_DN45594_c4_g1) (Fig. 9). In addition, genes encoding anthocyanidin reductase (TRINITY_DN52696_c0_g1) and hypothetical protein (TRINITY_DN45901_c1_g1) were the targets of the *MYB* gene (TRINITY_DN42931_c2_g2) (Fig. 9). These results suggest that the candidate TFs highlighted above may be involved in the regulation of secondary metabolism-related genes in the response of stylo to Mn toxicity.

### Validation of RNA-seq data using qRT-PCR

**Fig. 10:**
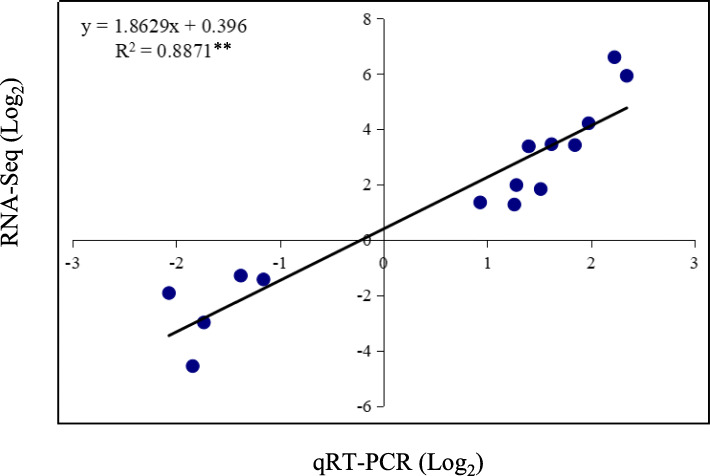
Correlation analysis of gene expression between transcriptome data and qRT-PCR results. Ten up-regulated and five down-regulated DEGs were selected for qRT-PCR analysis. Transcriptome data were plotted against data from qRT-PCR. Data are presented on a log_2_ scale

To confirm the RNA-seq data, 15 DEGs involved in secondary metabolism and TFs were further selected for qRT-PCR analysis (Fig. 10). These DEGs included 10 up-regulated and five down-regulated genes. The results showed a significant correlation (R^2^ = 0.887, *p* < 0.01) between the RNA-seq data and qRT-PCR results (Fig. 10), suggesting the reliability of the RNA-seq data.

### Changes in secondary metabolite levels under Mn stress

**Fig. 11:**
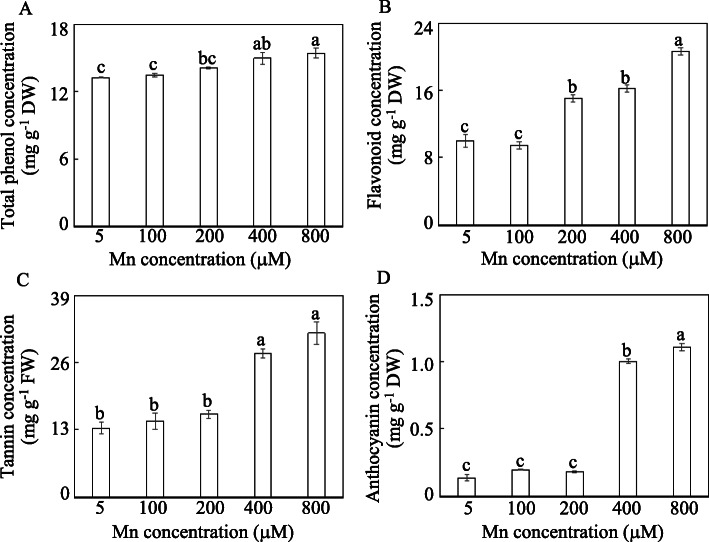
Concentrations of secondary metabolites in stylo leaves subjected to different Mn treatments. **a** Total phenols. **b** Flavonoids. **c** Tannins. **d** Anthocyanins. Thirty-day-old stylo plants were treated with 5 to 800 μM MnSO_4_ for 10 d. Values are the mean of three replicates with standard error bars. Different letters represent significant differences at *P* < 0.05

Subsequently, the concentrations of four secondary metabolites, including total phenols, flavonoids, tannins and anthocyanidins, in stylo leaves treated with various Mn concentrations were further detected. Compared to their respective controls, the levels of total phenols, tannins and anthocyanidins in stylo leaves increased with 400 and 800 μM Mn treatments, while the concentrations of flavonoids increased with Mn treatments increasing from 200 to 800 μM, peaking at 800 μM (Fig. 11). Furthermore, the concentrations of total phenols, flavonoids, tannins and anthocyanidins in stylo leaves subjected to 400 μM Mn treatment were increased 1.1-, 1.6-, 2.1- and 7.4-fold, respectively, compared with their controls (Fig. 11). These results suggest that secondary metabolism may play a key role in stylo’s response to Mn toxicity.

## Discussion

Although Mn is a trace element required for plant growth, Mn phytotoxicity generally occurs, especially in acid soil regions worldwide [8, 9]. Mn toxicity not only causes adverse impacts in plant cells but also threatens human health [27, 28]. In recent decades, genome-wide identification of metal responsive genes through transcriptomic techniques has been conducted to investigate the molecular mechanisms of plant adaptation to heavy metal stresses [29–31]. However, the transcriptome profiles of plants in response to Mn toxicity have not been well documented. In this study, the molecular responses of stylo to Mn toxicity were investigated through integration of physiological and transcriptomic analyses, providing potential Mn-tolerant strategies in plants for further study.

### Response of stylo to Mn toxicity

Stylo growth was inhibited by high Mn stress, as reflected by the inhibition of leaf chlorophyll concentrations and plant dry weight, especially under 400 and 800 μM Mn treatments (Figs. 1 and 2). Similarly, decreases in plant growth are also observed in ryegrass (*Lolium perenne*), white clover (*Trifolium repens*) and polish wheat (*Triticum polonicum*) under Mn toxicity [32, 33]. Stylo growth inhibition might be due to the overproduction of ROS, as indicated by the accumulation of O_2_^−^ and MDA (Fig. 1), which was supported by the results obtained from a previous study in stylo [11]. A considerable increase in oxidative stress is also found in polish wheat leaves under Mn toxicity, inhibiting shoot and root growth [33].

RNA-seq analysis was further conducted to identify DEGs in stylo’s response to Mn toxicity. The identified DEGs can be classified into numerous pathways (Fig. 4), suggesting various adjustments in stylo’s response to Mn toxicity. Among them, a large number of excess Mn-regulated genes were categorized as defense response genes, including homologous genes encoding antioxidant enzymes, such as POD and APX (Additional file 6: Table S5). This finding is consistent with a previous study demonstrating that homologous genes encoding antioxidant enzymes are up-regulated in stylo and citrus (*Citrus grandis*) in response to Mn toxicity [11, 34]. Furthermore, excess Mn up-regulation of *POD* and *APX* transcripts may result in increasing POD and APX activities in stylo leaves subjected to excess Mn toxicity (Fig. 3 and Additional file 6: Table S5), suggesting that these antioxidant enzymes may participate in ROS scavenging in stylo. In addition, 8 out of 10 genes encoding homologs to glutathione S-transferase (GST) were up-regulated by Mn toxicity (Additional file 6: Table S5). As a cellular detoxification enzyme, GST protects plants from oxidative damage [35, 36]. Homologous genes of *GST* play protective roles in plants against different stresses. For instance, the expression of *OsGSTLs* is enhanced by arsenic (As) stress in rice, and overexpression of *OsGSTL2* increases tolerance to As, cadmium (Cd) and chromium (Cr) stresses in transgenic *Arabidopsis* [35].

Moreover, other DEGs belonging to defense response related proteins, such as pathogenesis-related proteins (PRs), disease-resistance proteins (DRPs), heat shock proteins (HSPs), chitinases, beta-1,3-glucanases (GLUs) and late embryogenesis abundant proteins (LEAs), were also identified in this study (Additional file 6: Table S5). Homologues of these genes are regulated by Mn or other metal stresses [11, 29, 30, 37, 38], suggesting that overlap between metal stress responses may be conserved in stylo and other plants.

### DEGs involved in secondary metabolism

A group of DEGs involved in plant secondary metabolism, such as phenylpropanoid, flavonoid and isoflavonoid biosynthesis processes, were identified in this study (Figs. 6 and 7), suggesting that secondary metabolism is regulated by Mn toxicity. In the phenylpropanoid pathway, for example, the expression of *PAL* was up-regulated by Mn stress in the transcriptome data (Fig. 7). *PAL* is a key gene that catalyzes phenylalanine to form cinnamic acid in the phenylpropanoid pathway, which plays important roles in plant stress tolerance [39, 40]. Heavy metal treatments, such as lead (Pb), increase the transcript level of *PAL* or its enzyme activity in soybean (*Glycine max*), lupine (*Lupinus luteus*) and tomato (*Lycopersicon esculentum*) [41, 42]. Accordingly, increased *PAL* transcript levels may enhance its enzyme activity in stylo’s response to Mn toxicity (Figs. 3 and 7). Furthermore, some genes encoding proteins involved in different steps in the phenylpropanoid biosynthesis pathway, such as CCR, CADs, COMTs, F6Hs and PODs, were all up-regulated in stylo subjected to Mn toxicity (Fig. 7). Similar results are found in proteomic analysis of stylo, in which a group of proteins associated with the phenylpropanoid metabolic pathway are up-regulated under excess Mn stress [11]. Therefore, these results strongly suggest that the phenylpropanoid biosynthesis process is activated in stylo to counteract Mn stress.

The flavonoid biosynthesis process is performed by a set of key genes encoding CHSs, CHIs, FLSs and F3,5H, which were found to be regulated by Mn toxicity in this study (Fig. 7). *CHS*, *CHI* and *FLS* transcripts are regulated by abiotic stresses, such as UV light, wounding and heavy metal toxicity [42–44]. For example, *CHS* is a key gene involved in regulating flavonoid biosynthesis; *CHS* expression is enhanced in Pb-exposed tomato [42]. Similarly, *CHI* expression is increased in pea roots (*Pisum sativum*) under Cd stress [45]. In addition, *ZmFLS1* in maize is induced by UV-B radiation, and high expression levels of *ZmFLS1* protect plants from UV-B stress [43]. In addition, DEGs belonging to isoflavonoid metabolism related proteins, such as HIDH, I2H, I7OMT and VRs, were also regulated by Mn stress (Fig. 7). Isoflavonoids are a predominant subclass of flavonoid metabolites that play critical roles in plant defense [46, 47].

As important osmotic regulators and ROS scavengers in plant stress tolerance, secondary metabolites are regulated by Mn stress in many plants, such as phenylalanine in *Populus cathayana* [13], lignin and flavonoid in rice [2], and phenol and callose in cowpea (*Vigna unguiculata*) [12]. Similarly, in this study, significant increases in secondary metabolites, including total phenols, flavonoids, tannins and anthocyanins, were observed in stylo leaves exposed to 400 and 800 μM Mn (Fig. 11). The accumulation of secondary metabolites might be attributed to the regulation of various genes, such as *PAL*, *CCR*, *CAD*, *CHS*, *CHI*, *FLS* and *HIDH* (Fig. 7). Cumulative results suggest that increasing gene expression can cause the accumulation of secondary metabolites. For example, increases in *CHS* and *PAL* transcripts are closely related to the increased levels of phenols, flavonoids and anthocyanins in tomato under Pb stress [42]. Furthermore, exogenous expression of *ZmFLS1* from maize increases anthocyanin accumulation in an *Arabidopsis fls1* mutant [43]. Although the exact roles of the candidate DEGs described above warrant further research, the results presented in this study strongly suggest that adjustment of secondary metabolism and corresponding gene expression might be important for stylo adaptation to Mn toxicity.

### DEGs related to transcription factors

In this study, the identification of 123 transcription factors suggested complex regulation in stylo’s response to Mn toxicity (Fig. 8 and Additional file 5: Table S4). Among those identified transcription factors, seven DEGs belonging to the C2H2 zinc finger transcription factor family were up-regulated by Mn toxicity in stylo (Additional file 5: Table S4). C2H2 transcription factors are implicated in low pH-stress and aluminum (Al) toxicity tolerance in many plants, such as AtSTOP1/2 in *Arabidopsis*, VuSTOP1 in rice bean (*Vigna umbellata*) and GmSTOP1s in soybean [48–51]. For example, *GmSTOP1*–*1/1–3* expression is enhanced by Al toxicity in soybean roots; complementation analyses of *GmSTOP1*s in an *Arabidopsis Atstop1* mutant suggest that GmSTOP1s play critical roles in Al tolerance [51]. Furthermore, it has been demonstrated that C2H2 transcription factors function in Al detoxification mainly through activation of Al-tolerant genes, such as gene encoding glutamate dehydrogenase 1 (GDH1), pectin methylesterase inhibitor (PMI), malic enzyme (ME), aluminum-activated malate transporter (ALMT), multidrug and toxic compound exclusion protein (MATE) and tonoplast dicarboxylate transporter (TDT) [48, 49, 51]; these homologous genes were also up-regulated by Mn toxicity in stylo (Additional file 3: Table S2), suggesting that the functions of C2H2 transcription factors may be conserved in Mn detoxification through regulation of downstream critical Mn-resistant genes. Interestingly, two DEGs, *CAD1*/*2*, which belong to the phenylpropanoid biosynthesis pathway, were predicted to be regulated by six C2H2 transcription factors (Fig. 9), suggesting that these C2H2 transcription factors may also play transcriptional regulatory roles in secondary metabolism in stylo during Mn exposure, which deserves further study.

Moreover, nine transcription factor genes belonging to the WRKY family were up-regulated by Mn toxicity in stylo (Additional file 5: Table S4). Although the function of WRKY transcription factor remains largely unknown in stylo’s response to Mn toxicity, WRKY46 in *Arabidopsis* a negative regulator of ALMT1; disruption of *WRKY46* results in increased malate exudation, conferring Al tolerance [52]. However, *WRKY46* expression in *Arabidopsis* is suppressed by Al toxicity [52], but *WRKY* gene transcripts in stylo were enhanced by Mn stress in this study (Additional file 5: Table S4), suggesting that there are other regulatory networks that are mediated by WRKY-mediated gene expression in stylo during Mn toxicity.

Additionally, 22 transcription factors belonging to the MYB family were identified in this study (Additional file 5: Table S4). Cumulative studies have demonstrated that MYB family transcription factors participate in the response to environmental stresses in plants [53]. For example, *MYB49* expression in *Arabidopsis* is induced by Cd treatment; overexpression of *MYB49* in *Arabidopsis* results in a significant increase in Cd accumulation, inhibiting primary root growth [54]. Furthermore, MYB49 interacts with the transcription factors bHLH38 and bHLH101, leading to the activation of *IRON*-*REGULATED TRANSPORTER1* and mediating Cd uptake [54]. In addition, *OsARM1*, which encodes the R2R3 MYB transcription factor, is induced by As treatment in rice. *OsARM1* knockout improves tolerance to As stress in rice through regulation of As transporters, thereby mediating As uptake and translocation [55]. Furthermore, in this study, the MYB transcription factor is probably involved in the regulation of secondary metabolic pathways by mediating the transcripts of genes belonging to HIDH, anthocyanidin reductase and hypothetical protein (Fig. 9). Thus, future efforts are required to investigate the functions of these transcription factors in stylo subjected to Mn toxicity.

### DEGs related to transporters

Different kinds of DEGs belonging to transporters possibly involved in Mn translocation, distribution and sequestration were identified in this study (Additional file 7: Table S6). Among them, a set of genes encoding homologs to ATP-binding cassette (ABC) transporters were regulated by Mn toxicity. Plant ABC transporters constitute a large superfamily and are implicated in the transport of a variety of substrates, including hormones, secondary metabolites and heavy metal ions [56]. It has been demonstrated that the transcripts of *OsABCG43*, belonging to the rice ABC transporter, is up-regulated by Cd treatment; overexpression of *OsABCG43* confers Cd tolerance in the Cd-sensitive yeast mutant *ycf1acr3*, suggesting that the role of OsABCG43 in sequestration of Cd into subcellular organelles (e.g., vacuoles) [57]. OsALS1, encoding a half-size ABC transporter in rice, is found to be localized at the tonoplast and is required for Al detoxification through sequestration of Al into the vacuoles [58]. A similar result has been reported in Arabidopsis that AtALS1 participates in Al sequestration [59]. Furthermore, studies on cyanobacterium (*Synechocystis*) also suggest the potential role of ABC transporter in Mn transport [60]. Therefore, genes encoding ABC transporters are probably involved in stylo leaves defense against Mn toxicity.

Furthermore, genes encoding cation/H^+^ exchangers were found to be up-regulated by Mn toxicity in this study (Additional file 7: Table S6). The cation/H^+^ exchanger plays important roles in the vacuolar accumulation of metals, such as Mn. For example, the cation/H^+^ exchanger in Arabidopsis, AtCAX2, is involved in conferring tolerance to Mn toxicity when it is heterologously expressed in a Mn-sensitive yeast mutant *pmc1vcx1cnb* or in tobacco (*Nicotiana tabacum*) via sequestration of Mn into vacuoles [61]. Similar functions have been assigned to AtCAX4 and AtCAX5, which localize to the vacuolar membrane [62, 63]. In addition, one gene encoding homologs to aquaporin protein, aquaporin TIP1–3-like, was also up-regulated by Mn toxicity in stylo leaves (Additional file 7: Table S6). Genes belonging to the aquaporin protein family are reported to be involved in plants response to metal stresses. For example, two genes encoding tonoplast-intrinsic protein (TIP) are regulated by Al toxicity in buckwheat (*Fagopyrum esculentum*) leaves [64]. It has been demonstrated that HmVALT, belonging to the tonoplast-intrinsic protein in Al hyperaccumulating hydrangea (*Hydrangea macrophylla*), is a tonoplast-localized Al transporter that involves in vacuolar Al transport; overexpression of *HmVALT* confers Al tolerance in *Arabidopsis* [65]. There results suggest the potential roles of this gene in vacuolar Mn transport.

In addition, a group of genes belonging to transporters for various substrates, including phosphate, potassium and copper, were found to be differentially regulated by Mn in stylo leaves (Additional file 7: Table S6). The balance between the accumulation of Mn and other abundant mineral elements is thought to be important for regulating Mn homeostasis in plants [33]. Thus, these DEGs related to transporters responsible for Mn translocation, distribution and sequestration are regarded to be critical for intracellular Mn detoxification in stylo, and their functions warrant further investigation.

## Conclusion

In conclusion, this study showed that stylo growth was inhibited by high Mn due to oxidative stress. Consistently, transcriptome analysis showed that a set of DEGs involved in regulating ROS scavenging and defense responses might contribute to stylo’s response to Mn toxicity. Furthermore, the identified DEGs associated with secondary metabolic pathways suggested that fine-tuned regulation of secondary metabolism might represent an adaptive strategy of stylo to cope with Mn toxicity. In addition, activation of various transcription factors may help stylo to tolerate Mn toxicity through transcriptional regulation. Taken together, this study not only reveals new insights into the molecular mechanisms underlying stylo’s response to Mn toxicity but also provides candidate genes that may be useful in the development of crop varieties with Mn adaptability through genetic engineering.

## Methods

### Plant growth and treatments

The stylo (*Stylosanthes guianensis*) genotype ‘TF303’ used in this study was provided by the Tropical Pasture Research Center, Institute of Tropical Crop Genetic Resources (TCGRI), Chinese Academy of Tropical Agriculture Sciences (CATAS), Hainan, China (voucher specimen deposited in the National Tropical Forage Germplasm Genebank, TCGRI, CATAS). The plant material was authenticated by Guodao Liu, TCGRI, CATAS. All experimental procedures were performed at the TCGRI, CATAS, Hainan, China (19°30′N, 109°30′E). After seed germination, stylo seedlings were transferred to a modified Hoagland nutrient solution (pH 5.8) containing 2500 μM KNO_3_, 2500 μM Ca (NO_3_)_2_, 400 μM NH_4_NO_3_, 250 μΜ KH_2_PO_4_, 500 μM MgSO_4_, 250 μM K_2_SO_4_, 5 μM MnSO_4_, 0.5 μM ZnSO_4_, 1.5 μM CuSO_4_, 0.09 μM (NH_4_)_6_Mo_7_O_24_, 23 μM NaB_4_O_7_ and 80 μM Fe-Na-EDTA [26]. After 30 d of normal growth, stylo seedlings were transplanted into fresh nutrient solution supplied with 5, 100, 200, 400 and 800 μM MnSO_4_ (pH 5.0). The background Mn level in the nutrient solution is 5 μM and thus used as the control [26]. After 10 d of Mn treatments, shoots and roots were harvested for further study. An individual hydroponic box containing four stylo seedlings served as one biological replicate. All treatments included three biological replicates.

### Analysis of chlorophyll concentrations

To analyze chlorophyll concentrations, approximately 0.05 g leaf sample was incubated in 10 mL of 80% (v/v) acetone for 6 h. The chlorophyll concentration was spectrophotometrically detected at 440, 645 and 663 nm using a UV-2010 spectrophotometer (Hitachi, Japan) and determined as previously described [26].

### Determination of Mn concentrations

To measure Mn concentrations, approximately 0.07 g dry shoot and root samples were transferred to a muffle furnace and burned at 600 °C for 10 h. Then, the sample was adequately extracted in 7 mL of 100 mM HCl. Mn concentrations in each sample were detected using atomic absorption spectroscopy [11].

### Detection of O_2_^−^ and MDA levels

O_2_^−^ levels in stylo leaves were detected using the histochemical method [66]. Leaves subjected to different Mn treatments were incubated in 0.25 mM NBT solution for 12 h. Samples were then decolorized in boiling ethanol for 6 h. O_2_^−^ levels were assessed in leaves based on the intensity of the blue insoluble formazan.

For MDA analysis, approximately 0.1 g samples were extracted in 1 mL of 10% trichloracetic acid (TCA). Homogenates were centrifuged at 12,000×*g* for 10 min. The supernatants were collected, and 1 mL supernatant was added to 1 mL of 0.5% thiobarbituric acid (TBA). The mixture was then incubated at 100 °C for 20 min. Then, homogenates were centrifuged at 12,000×*g* for 10 min. The MDA concentration was detected as previously described [67].

### Analysis of electrolyte leakage and *F*v/*F*m

Stylo leaves treated with various Mn concentrations were separately immersed in 10 mL of deionized water in a glass test tube overnight at room temperature. The initial conductivity (IC) of the solution was detected using a conductimeter (INESA Scientific Instrument Co., Ltd., Shanghai, China). Total conductivity (TC) of the solution was detected after boiling for 15 min. Relative electrolyte leakage was expressed as the percentage of the initial conductivity (IC) to the total conductivity (TC). *F*v/*F*m values of stylo leaves were measured using a pulse-modulated fluorometer Model FMS-2 (Hansatech Instruments Ltd., UK) according to the manufacturer’s instructions.

### Analysis of enzyme activity

To determine POD activity, approximately 0.1 g leaf samples from different Mn treatments were extracted in 2 mL of 50 mM phosphate buffer (pH 7.8) at 4 °C. The extracts were centrifuged at 12,000×*g* for 15 min at 4 °C, and the supernatants were recovered for POD assay [66]. APX was extracted by grinding 0.1 g leaves in 2 mL of 50 mM phosphate buffer (pH 7.0) supplied with 1 mM AsA and 1 mM ethylenediaminetetraacetic acid (EDTA) at 4 °C. After centrifugation, the supernatants were collected for APX activity assays [66].

To detect PPO activity, approximately 0.1 g leaf samples were extracted in 1 mL of extraction buffer obtained from commercial assay kits (Solarbio Science and Technology Co., Ltd., Beijing, China) at 4 °C. The extracts were centrifuged at 12,000×*g* for 15 min at 4 °C, and supernatants were collected for PPO activity assessment at 410 nm. PPO activity was calculated according to the manufacturer’s protocol. To analyze PAL activity, 0.1 g leaf samples were extracted in 0.9 mL of extraction buffer obtained from commercial assay kits (Nanjing Jiancheng Bioengineering Institute, Jiangsu, China) at 4 °C. After centrifugation, the supernatants were used to determine PAL activity at 290 nm according to the protocol.

### Transcriptome analysis of stylo leaves

RNA sequencing was performed by Annoroad Gene Technology Co., Ltd. (Beijing, China). Total RNA of stylo leaves subjected to control (5 μM) and Mn toxicity (400 μM) treatments was isolated using TRIzol reagent (Invitrogen, Carlsbad, CA, USA). RNA integrity and purity were analyzed using an Agilent 2100 RNA Nano 6000 Assay Kit (Agilent Technologies, Palo Alto, CA, USA). Sequencing libraries were generated using the NEBNext Ultra Directional RNA Library Prep kit (NEB, Beverly, MA, USA). The cDNA libraries of the three biological replicates were obtained using an Illumina Hiseq™ 4000 platform (Illumina, San Diego, CA, USA). PE150 was used as the sequencing program.

Raw image data were transformed to raw reads using the Consensus Assessment of Sequence and Variation (CASAVA, v.1.8.2) base recognition program. The raw reads were stored in FASTQ files. High-quality clean reads were obtained by filtering out adaptor sequences and low-quality reads. The obtained clean reads were further assembled into unigenes using Trinity software (version 2.4.0) [68]. The clean reads were mapped back to the assembled contigs through Bowtie2 v.2.2.3 software [69]. For annotation, all unigene sequences were aligned by BLAST search (E-value<1e-5) against the NCBI non-redundant protein sequences (Nr), NCBI non-redundant nucleotide sequences (Nt), Universal Protein (UniProt), Gene Ontology (GO), the Clusters of Orthologous Groups of protein database (COG), the Functional Annotation (eggNOG), the KEGG database and the Protein Family (Pfam).

The transcript abundance of each gene in each sample was calculated and represented by the reads per kilobase million mapped reads (RPKM) value using RNA-seq data with expectation maximization (RSEM) [70]. DESeq2 was used to calculate the differences in the expression abundance between 5 and 400 μM Mn treatments [71]. Genes with *q* < 0.05 and |log_2_(fold change)| ≥1 were considered DEGs. Subsequently, DEGs were extracted for GO and KEGG enrichment analysis [72, 73]. Protein-protein interaction network analysis for the identified DEGs was performed using STRING (version 11.0) (https://string-db.org/). The interaction networks were constructed using Cytoscape (version 3.8.0) (https://github.com/cytoscape/cytoscape) with default settings. Nodes and edges indicate genes and interactions, respectively. The sequencing data were deposited in the National Center for Biotechnology Information (NCBI) Gene Expression Omnibus under GEO series number GSE147431.

### Quantitative real-time PCR (qRT-PCR) analysis

Total RNA from leaves was extracted using the TRIzol reagent (Invitrogen, USA) according to the manual. The first cDNA strand was synthesized from 2 μg of DNase I-treated RNA using oligo d(T), dNTPs, RNase inhibitor and reverse transcriptase in the HiScript III cDNA synthesis kit (Vazyme, China) according to the protocol. qRT-PCR analysis was performed in a 20 μL volume containing 2 μL 1:50 diluted cDNA, 0.2 μM gene primers, 6.4 μL ddH_2_O and 10 μL SYBR Green Master mix (Vazyme, China). The reaction was performed on a QuantStudio™ 6 Flex Real-Time System (Thermo Fisher Scientific, Waltham, MA, USA). qRT-PCR analysis conditions were as follows: 95 °C for 1 min, 40 cycles of 95 °C for 15 s, 58 °C for 15 s and 72 °C for 30 s. qRT-PCR primers of genes are listed in Additional file 8: Table S7. The housekeeping gene *SgEF-1a* (Accession No. JX164254) was used as an endogenous control to calculate the relative gene expression as the ratio of the transcript of the candidate gene to that of *SgEF-1a* [26]. Gene expression analysis included three biological replicates.

### Determination of secondary metabolite concentrations

The concentrations of secondary metabolites, including total phenols, flavonoids, anthocyanins and tannins, in stylo leaves exposed to various Mn treatments were analyzed using commercial assay kits (Nanjing Jiancheng Bioengineering Co., Ltd., Jiangsu, China). To detect total phenols, flavonoids and anthocyanins, 0.1 g dry leaf samples were extracted and measured as previously described [66]. For tannin assays, approximately 0.1 g fresh leaf samples were extracted in 1 mL of ddH_2_O. The concentration of tannins was detected according to the manufacturer’s protocol.

### Statistical analysis

Microsoft Excel 2003 (Microsoft Company, USA) was used in the data analysis. One-way analysis of variance (ANOVA) was performed using SPSS software, version 13.0 (SPSS Institute, Chicago, USA).

## Supplementary Information

**Additional file 1: Figure S1.** Effects of different Mn treatments on relative electrolyte leakage, *F*v/*F*m, plant height and total protein content.

**Additional file 2: Table S1.** Summary of stylo leave transcriptomes in control (5 μM) and Mn toxicity (400 μM) libraries.

**Additional file 3: Table S2.** Information of DEGs identified in stylo leaves subjected to control (5 μM) and Mn toxicity (400 μM) treatments.

**Additional file 4: Table S3.** DEGs involved in secondary metabolism.

**Additional file 5: Table S4.** Transcription factors responding to Mn toxicity.

**Additional file 6: Table S5.** DEGs involved in defense responses.

**Additional file 7: Table S6.** DEGs related to transporters.

**Additional file 8: Table S7.** Primers used for qRT-PCR analysis.

## Data Availability

The datasets for this study can be found in the NCBI Gene Expression Omnibus under GEO series number of GSE147431.

## Abbreviations

APX: Ascorbate peroxidase
CAD: Cinnamyl-alcohol dehydrogenase
CALDH: Coniferyl-aldehyde dehydrogenase
CCR: Cinnamoyl-CoA reductase
CHI: Chalcone isomerase
CHS: Chalcone synthase
COMT: Caffeoyl-CoA O-methyltransferase
CSE: Caffeoylshikimate esterase
DEGs: Differentially expressed genes
F3’5’H: Flavonoid 3,5-hydroxylase
F6H: Feruloyl-CoA 6-hydroxylase
FLS: Flavonol synthase
GLU: Beta-glucosidase
GO: Gene Ontology
GST: Glutathione S-transferase
HI4OMT: 2,7,4-Trihydroxyisoflavanone 4-O-methyltransferase/isoflavone 4-O-methyltransferase
HIDH: 2-Hydroxyisoflavanone dehydratase
I2H: Isoflavone 2-hydroxylase
I7OMT: sofilavone-7-O-methyltransferase
KEGG: Kyoto Encyclopedia of Genes and Genomes
MDA: Malondialdehyde
NBT: Nitroblue tetrazolium chloride
PAL: Phenylalanine ammonia-lyase
POD: Peroxidase
PPO: Polyphenol oxidase
qRT-PCR: Quantitative real-time polymerase chain reaction
RNA-seq: RNA sequencing
ROS: Reactive oxygen species
SOD: Superoxide dismutase
SPL19: Serine carboxypeptidase-like 19
TFs: Transcription factors
VR: Vestitone reductase
