# Supplementary material for: Physiological and transcriptomic analyses reveal the roles of secondary metabolism in the adaptive responses of Stylosanthes to manganese toxicity
Source: BMC Genomics. 2020 Dec 3;21:861. doi: 10.1186/s12864-020-07279-2 (PMC7713027; doi:10.1186/s12864-020-07279-2)
Supplement: Supplementary file 1 — Additional file 1: Figure S1. Effects of different Mn treatments on relative electrolyte leakage, Fv/Fm, plant height and total protein content. [file 12864_2020_7279_MOESM1_ESM.pdf]

Additional file 1: Figure S1

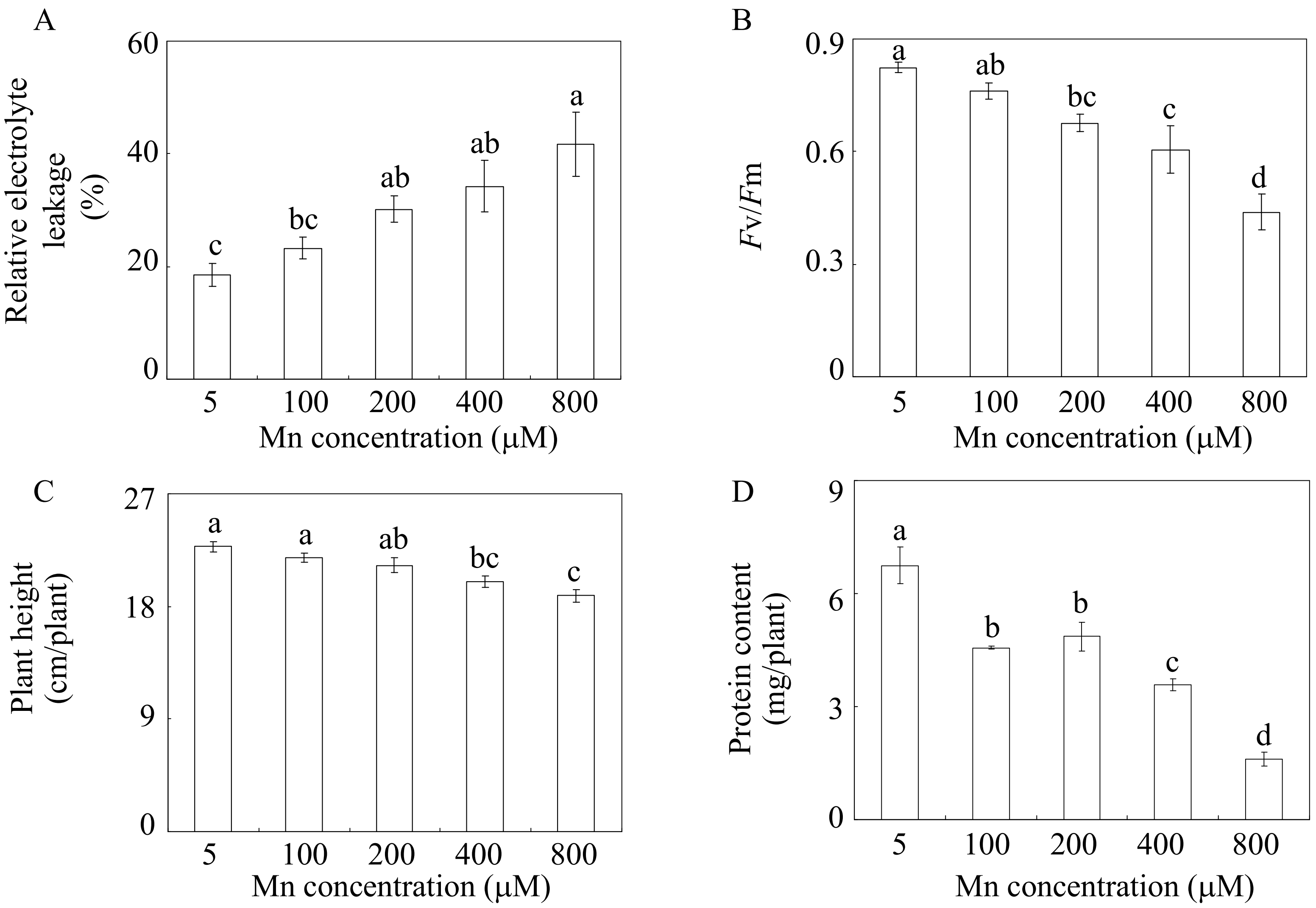

Additional file 1: Figure S1. Effects of different Mn treatments on relative electrolyte leakage, *F*v/*F*m, plant height and total protein content. (a) Relative electrolyte leakage. (b) *F*v/*F*m values. (c) Plant height. (d) Total protein content. Thirty-day-old stylo plants were treated with 5, 100, 200, 400 and 800 μM $MnSO_4$ for 10 d. Values are the mean of three replicates with standard error bars. Different letters represent significant differences at $P<0.05$.
